# An Optimized Load Balance Solution for Multi-homed Host in Heterogeneous Wireless Networks

**DOI:** 10.3390/s19122773

**Published:** 2019-06-20

**Authors:** Mohamed Naeem, Hussein M. ELAttar, Mohamed Aboul-Dahab

**Affiliations:** 1 Computer networks and data center-Cairo, Arab Academy for Science, Technology and Maritime Transport, Cairo 11799, Egypt; 2 Department of Electronics and Communications Engineering. Arab Academy for Science, Technology and Maritime Transport, Cairo 11799, Egypt; hattar@aast.edu (H.M.E.); mdahab@aast.edu (M.A.-D.)

**Keywords:** HetNet, MADM, MCDM, AHP, QoS, application preferences, network selection, multihoming, user-centric, load balance, utility connection equation

## Abstract

The imminent wireless technologies are demanding fusion of several networks with diverse technologies. A convenient user device for such an environment is the multihomed host, which is capable of making use of simultaneous multiple connections of heterogeneous networks and smartly distributing/receiving data among them. The aim of this work is to develop a mechanism for assessing the multihoming concept and to propose a system model for increasing its applicability. The proposed model provided a novel user-centric scheme for multihoming for multi-radio access technologies (multi-RAT). It is considered an evolution of traffic offloading for gaining more capacity, higher data rates, and real-time services. This work assumes data classifications, evaluates and ranks the available connections, and utilizes the application data in an unequal load balance scheme. This is achieved by analyzing the performance of three of the most widely used alternative-choice for multiple attributes decision making (MADM) methods. The analytical hierarchy process (AHP), along with a utility equation, is applied to the system model for unequal load balance solution. The proposed model is targeting an energy efficient mechanism which satisfies application needs. Moreover, it reveals an efficient transmission mechanism for a better quality of service, traffic management, and availability solutions. The simulation results show that the proposed system surpassed its former counterparts.

## 1. Introduction

The fifth generation (5G) cellular network is supposed to be an integrative network guaranteeing a seamless user experience. This is done to satisfy a more demanding capacity, higher data rates, and real-time services between massive numbers of connected devices. With the rise of new communications services such as machine-to-machine M2M, device-to-device D2D, IoT, etc., there is a need for cooperation among various operating systems to take full advantage of the available accessible bandwidth.

Nowadays, world countries are racing to develop the 5G network infrastructure as a heterogeneous network (HetNet). While the homogeneous network relies only on one type of communication technologies, the heterogeneous network integrates different ones [1]. The heterogeneous network aims at offering better connection speed and coverage. The development cycle calls for applying the emerging technologies in 5G, which will inevitably become the yardstick for modern societies [2,3,4,5]. This development encourages the network operators to implement a small cell network formed by the availability of multi-radio access technologies (multi-RAT). This allows multi-RAT capable devices to connect to different wireless networks such as wireless fidelity (Wi-Fi) and long term evolution protocol (LTE) [6]. 

Heterogeneous networks, such as cellular systems and Wi-Fi access points, are configured so that data between accessible networks may be offloaded. However, in modern wireless networks, simultaneous connections to more than one network were lately standardized under “dual connectivity” in the 3GPP Release 12 [7] for the ability to connect to dual networks in the same time. Sending and receiving traffic over multiple networks at the same time is called “multihoming”. This is supposed to be an evolution of traffic offloading, increasing the utilization of the available resources. Thus, the multi-homed device supports multiple connections to a variety of networks, which may be heterogeneous or homogeneous.

In the past decades, applications have played an important role in pushing the multi-homing process in terms of the device manufacturers and network operators’ sides. They are still reforming to meet the increasing demands for the excessive number of users and devices [8]. Recently, an applications forecast revealed that the massive application data traffic to be acquired globally is up to 23 exabyte [9]. This huge amount of data turns out to be even more problematic because they require more bandwidth and less processing time. Most of the research in this field aims at solving this problem. Moreover, the experience of using the applications is witnessed to be enhanced within the multiplicity provided by the networks and end-user devices. Lack of the capability of the end user devices is missing the opportunity to improve. For those reasons, many research, including this paper, is sticking to provide a solution.

The solution may be addressed by network operators as a network-centric methodology or by the end user device as a user-centric methodology [10]. Network-centric methodology in network infrastructure includes devices, routers, and servers. Multihoming for routers is usually performed by border gateway protocol (BGP), which is an exterior gateway routing protocol. However, multi-homing for servers is maintained by considering homogeneous connections only [11]. A well-known problem with the network-centric solution is that it does not take into account the network orchestration. Orchestration provides the mechanism for all network elements in order to perform as one team plays for the same target as a team player, not as a solitary node. One way to overcome this problem is provided by the use of software-defined networking (SDN) as a network-centric methodology. Unfortunately, this approach has some shortcomings that are not limited to the necessity for technical expertise and the centralized control of policies [12]. 

On the other hand, the user-centric methodology is maintained on the user terminal to engage directly with the multiple connections. Multihoming for user devices is widely considered in providing everlasting, ubiquitous access and reliability. User devices do not have the required intelligence to make use of multiple connections and operate with only a single preferred connection while maintaining other remaining connections as standby [6]. There are techniques that have been developed to solve this problem such as handover, mobility, bandwidth aggregation, multiplexing, and load balancing [13].

The load-balance is implemented in HetNet wireless networks by the dynamic optimization of the utility function of the split ratio of data among multiple access networks without considering the type of application or the needed quality of service QoS [14,15,16,17]. Up to the best of our knowledge, no work considered the possibility of data distribution by unequal load balance with multiple access simultaneously while considering the application type as a leading perspective. Simultaneous multiple access is the newest concept in HetNet, which can as well be called “multihoming”.

Motivated by the need of 5G networks to deliver new services with higher data rates, the objective of this paper is to propose a framework for evaluating the multihoming principle and introducing a system for improving its applicability for devices with numerous interfaces. This paper tackles the load balance issue. The solution for load balance requires the ranking of available multiple connections. This is achieved using algorithms for multiple attributes decision making (MADM) [18].

The MADM can frequently be depicted as the operation of selecting one from many available options, or placement decisions. This paper studies the performance and compares three of the most remarkable MADM techniques in alternative-choice-making. This is to suggest that the most appropriate solution technique for the multi-homed host (MH), is to send the application data traffic in an unequal cost load balance distribution while matching both the application categories and connection parameters. This aims to better optimize the utilization of the available resources. These techniques are the: simple additive weighting (SAW) method, technique for order preference by similarity to ideal solution (TOPSIS) method and analytical hierarchy process (AHP) method [19].

The proposed model is considered a general solution for end-user devices and terminals. Simulation results are carried out to investigate the validation and performance of the proposed model. Simulation results aim to reveal that the proposed model can achieve the required objective, which is transmitting the data traffic per application preference over the available network connections in an unequal cost load balance scheme instead of offloading application data based on the ranking of connections.

This paper is organized as follows: Section 2 presents related works and problem statement of the load balance issue. Section 3 presents the proposed system model and provide the connection utility equation. Section 4 presents the simulation and analysis of the proposed model. Section 5 provides insight into future work and Section 6 concludes the paper.

## 2. Related Work

The related work section is divided into two subsections. The first one surveys some of the related efforts, and the second section provides a study to the multiple criteria decision making (MCDM) methods that are related to the proposed solution.

### 2.1. Multi-Homed Mobile Host Solution

**Figure 1:**
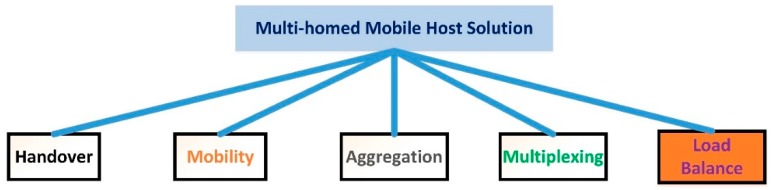
Multi-homed host solutions.

This section reviews the solutions related to the issue of MH in HetNet [13]. There are numerous studies to investigate this issue and solutions are proposed to perform handover. Some of these solutions focus on mobility, where others focus on aggregation. However, some solutions are based on multiplexing, and there are a variety of load balance solutions available in the literature. The solutions of MH in HetNet are illustrated in Figure 1.

#### 2.1.1. Handover Based Methods

The handover methods are based on traffic offloading between available network connections. Handover based on fuzzy logic has been devised in order to provide an approximation for multi-criteria interface selection. However, this technique requires high processing [20,21]. Distributed fuzzy engines have been proposed to provide lower processing overhead [22,23,24]. On the other hand, both energy saving and quality of service (QoS) parameters have been committed as a metric for the handover of application traffic [25,26]. The concept of best performance based on avoiding low rate links and favorite best rate link is given in [27,28]. The method introduced by scheduling application data based on the optimization technique has the advantage of a load balance of application traffic as given in [29]. Some authors have driven the further development of handover solution as a load balance over multiple connections as in [30], based on utilizing TOPSIS algorithm as an application based handover solution, and in [31], as an offloading of application data based on adapted connections ranking using AHP.

#### 2.1.2. Mobility Based Methods

Mobility is an MH solution aims at seamlessly moving between connections. The author in [32] employed an end-to-end mobility methodology which prescribes the use of control for user management, service management, and device control. A number of existing studies have examined the host identity protocol (HIP) to make a better mobility solution through several techniques such as: naming convention [33], using an ID between transport and network layer [34], robust path connection [35] and as a multi-path transmission [36]. 

#### 2.1.3. Aggregation Based Methods

Aggregation is an MH solution used either as a generic or as a specific solution. One generic solution is to address the end terminal application by providing a middle way between software applications and the operating system with duplicate transmission of data traffic [37,38]. One method employed in [39] is to aggregate the overall data. Some authors have driven a specific solution with the further development of a scheduling process to better transmission of application data over the available connections [40].

#### 2.1.4. Multiplexing Based Methods

Multiplexing of application data over multiple connections based on layer four protocols is a common MH strategy. There are several methods available in the literature that have examined Multiple Protocol Transmission Control Protocol (MPTCP) to provide a multiplexing solution for TCP traffic using division and sub-flow for the data under transmission [41]. One of these methods are based upon asymmetric routing and is characterized by the advantage of enhancing the time-sensitive application performance [42]. Another method is based on using security mechanisms and is found beneficial in data multiplexing and security [43]. In [44] the usage of data multiplexing as a redundant flow transmission has resulted in improving the application data integrity.

#### 2.1.5. Load Balance Based Methods

Load balance is an MH solution that aims at sending data over multiple connections in a fair distribution form, as either equal or unequal distributions [45]. Several techniques have been proposed to perform load balance of the application data, some of which have been focusing on distributing data traffic, others on multipath routing.

Data traffic distribution by distributing traffic threads have been recognized in [46]. Load balance based on distributed data chunks is given in [47]. The use of scheduler to load balance application data flow is explained in [48]. Using dynamic scheduling provides better performance by dynamic processing and allocation of application data [49]. The usage of scheduling based on QoS which provides better evaluation is given in [50,51].

The multipath routing is a method to achieve load balance and fair distribution. One of its techniques is based upon the usage of full path delay as a metric for simultaneous data transmission [52]. Another solution is adopting active scheduling for bandwidth aggregation of multiple interfaces [53]. For better multipath routing of video applications, one may use the frame-level control [54]. The usage of a scheduler for multi-path video transmission is proposed in [55]. Enhancing the processing delay is done by using a multipath routing mechanism [56], while it is optimized using full path content routing [57]. Another optimization is based on using a propping mechanism [58]. Multi-path routing is maintained by alternative approaches such as the internet protocol (IP) binding [59]. Solving the re-transmission issue by the usage of the multi-path data transmissions is given in [60]. Utility function has been devised to achieve multipath routing [61]. Multipath routing is in need to be controlled, and an approach has been introduced by applying the control plane processing [62]. The multipath gateway is another way to control the multipath routing [63]. The usage of virtual ID routing protocol (VIRO) for maintaining the multipath routing is introduced in [64].

Considering QoS to perform load balance is solved in several ways [65]. One way is the usage of the routing table for load balance over QoS verified paths as a host-based model [66]. Another way is the genetic crossover and mutation based on the overall QoS path parameters are used for path selection [67]. A third way is by achieving both mobility and load balance according to the application awareness [68]. Most techniques using multiple connections adopted QoS for more efficient performance. One common approach for all techniques is the load balance of application data either through the session identification, application ports, address mobility, and distribution of application data. There is a tradeoff between the application performance and the proposed load balance solutions. 

A challenging problem which arises in this domain is to provide a load balance solution by considering all application types. Only a few studies have shown an interest in providing a general solution. These will be considered for comparison with the proposed solution. The previous efforts were distinguished in terms of ranking the available connections and provide load balance by offloading application data over them, but they have issues related to the improvement of some applications performance more than others [30,31].

Compared to the previous related work, we have a different method, using an unequal cost load balance approach. This has been proposed to surmount the problems caused by the improved performance of certain applications at the expense of other applications. We are proposing a system model that is considered a general solution for concurrent multiple communication of applications data using unequal cost load balance technique.

### 2.2. Methods 

The heterogeneous multi-access network selection problem is considered as an NP-Hard problem [69,70]. In order to pick the most appropriate network in terms of quality of service, several challenges need to be considered. Three main challenges can be mentioned to solve the problem of network selection and traffic load balancing. The first challenge is the suitable criteria that should be used for data distribution among networks while considering the QoS parameters. The second challenge is determining a suitable algorithm that enables each criterion to be weighted. The third challenge is ranking the optimal networks for these criteria.

The load balance solution requires a ranking of the available multiple connections. This is performed using the MADM algorithm, which is a category of MCDM. The MADM can be commonly portrayed as the procedure of choosing one from many accessible choices, or positioning choices, in view of many criteria, which normally have an alternate essentialness. This section examines some of the most notable MADM methods in alternative decision making for providing a suitable solution for an issue with multiple criteria, such as the SAW, AHP, and TOPSIS methods. These methods are explained in the following subsection. 

#### 2.2.1. Simple Additive Weighting 

Created by means of MacCrimon in 1968, SAW is also known as the weighted linear combination, scoring method, or weighted sums [71]. SAW applies the precept of weighted average. In this technique, a scaled cost is assigned for each choice with the aid of an attribute. The basic thought of the SAW technique is to discover the weighted sum of overall performance rankings on each choice of all attributes. The SAW method requires the method of normalizing the decision matrix (R) to a scale similar to all present choice ratings [72].

(1) $$r_{ij}\;=\{\begin{array}{c} \frac{X_{ij}}{\max X_{ij}}\;,\;if\;j\;is\;a\;benefit\;attribute\;\\ \frac{\min X_{ij}}{X_{ij}},\;if\;j\;is\;a\;cost\;attribute\;\;\;\;\;\;\; \end{array}$$

where $r_{ij}$ is normalized performance rating from alternative $A_{i}$ on attribute $C_{j}$, *i* = 1, 2... *m*, and *j* = 1, 2.., *n*.

The preference value for each alternative ($V_{i}$) is given according to:

(2) $$V_{i}=\sum_{j=1}^{n}w_{j}r_{ij}$$

whereas:

$A_{i}$ = alternative
$C_{j}$ = criteria
$w_{j}$ = weight preference
$V_{i}$ = preference value for each alternative
$X_{ij}$ = alternate value of each criterion.

A larger value of $V_{i}$ shows that $A_{i}$’s choices are preferred. As for the standards, they are divided into two categories, namely for positive values included in the criteria of income and the negative cost protected in the value criteria. The following is the SAW method algorithm: 

1. Normalize the decision matrix by calculating the normalized performance rating ($r_{ij}$) value of alternative $A_{i}$ on criterion $C_{j}$ by using Equation (1). 

2. The result of a normalized performance rating ($r_{ij}$) values a normalized matrix as in Equation (3).

(3) $$R\;=\;\left[\begin{array}{ccc} R_{11} & \cdots & R_{1j}\\ \vdots & \ddots & \vdots \\ R_{i1} & \cdots & R_{ij} \end{array}\right]$$

3. The final result of the preference value ($V_{i}$) is bought from the summing of the matrix row element matrix (R) with the corresponding weight of choice (W) of the matrix column component (W). The value of preference uses Equation (2). Greater $V_{i}$ score calculations indicate that the alternative $A_{i}$ is the best alternative. 

#### 2.2.2. Technique for Order Preference by Similarity to Ideal Solution

TOPSIS is a MADM method created by Hwang and Yoon in 1981, the TOPSIS method for choosing alternatives that simultaneously had the lowest distance from the ideal solution and the outmost distance from the ideal solution [73,74,75]. A positive ideal solution maximizes the benefit criteria and minimizes the cost criteria, while the ideal negative solution maximizes the cost criteria and minimizes the benefit criteria. To apply this technique, attribute values must be numerical, monotonically increasing or decreasing, and having equivalent units.

The steps in completing a MADM case with TOPSIS is as follows: 

1. Make a normalized decision matrix using:

(4) $$r_{ij}=\;\frac{X_{ij}}{\sqrt{\sum_{i=1}^{m}X_{ij}{}^{2}}}$$

where:

$r_{ij}$ = the normalized value of the decision matrix
$X_{ij}$ = the original value of the decision matrix

2. Create a normalized weighted decision matrix using:

(5) $$y_{ij}=w_{j}r_{ij}$$

where:

$y_{ij}$ = a weighted normalized decision matrix
$w_{j}$ = weighting against criterion *i*
$r_{ij}$ = the normalized value of the decision matrix

3. Determine the matrix of positive ideal solutions and the ideal negative solution matrix by using:

(6) $$\;A^{+}=\left(y_{1}{}^{+},\;y_{2}{}^{+},\ldots \ldots ,\;y_{n}{}^{+}\right);\;\;A^{-}\;=\;\left(y_{1}{}^{-},\;y_{2}{}^{-},\ldots \ldots ,\;y_{n}{}^{-}\right);$$

(7) $$y_{1}{}^{+}=\{\begin{array}{c} max_{iyij}\;,\;if\;j\;is\;a\;benefit\;attribute\;\\ min_{iyij}\;,\;\;if\;j\;is\;a\;cost\;attribute \end{array}\;y_{1}{}^{\_\;}=\{\begin{array}{c} max_{iyij}\;,\;if\;j\;is\;a\;benefit\;attribute\;\\ min_{iyij}\;,\;\;if\;j\;is\;a\;cost\;attribute \end{array}$$

where:

$A^{+}$ = positive idea solution $A^{+}$ matrix
$A^{-}$ = matrix solution negative idea $A^{-}$
$y_{1}{}^{+}$ = $max_{iyij}$ if *j* is a benefit attribute *(benefit)*
$max_{iyij}$ = if j is a cost attribute *(Cost)*
$y_{1}{}^{-}$ = $min_{iyij}$ if *j* is a benefit attribute *(benefit)*
$min_{iyij}$ = if *j* is the cost attribute *(Cost)*

4. Determine the distance between the value of each alternative with the matrix of positive ideal solutions and the ideal negative solution matrix using:

(8) $$D_{i}{}^{+}=\sqrt{\sum_{j=1}^{n}{\left(y_{i}{}^{+}-\;y_{ij}\right)}^{2}}$$

(9) $$\;D_{i}{}^{-}=\;\sqrt{\sum_{j=1}^{n}{\left(y_{ij}-\;y_{i}{}^{-}\right)}^{2}}$$

where:

$D_{i}{}^{+}$ = distance to a positive ideal solution
$D_{i}{}^{-}$ = distance to the ideal solution negative

5. Determine the preference value for each alternative by using:

(10) $$V_{i}\;=\;\frac{D_{i}{}^{-}}{D_{i}{}^{-\;}-\;D_{i}{}^{+}}$$

The preference value is the final value used to rank all previously assessed alternatives. The preference value of an alternative is the ratio between the distance from the ideal solution and the amount of distance to the ideal positive solution. If the value $V_{i}$ represents the greatest value, it indicates that the alternative *Ai* has been appropriately selected.

#### 2.2.3. Analytical Hierarchy Process 

Developed by Thomas Saaty in the 1970s, The AHP ranking process is varying for the application class. The AHP numerical process starts by constructing the pairwise comparison matrix of the application class. The matrix is filled by numerical values that are formed by the importance of each row element to its opposite column element. Numerical values are maintained in a relative importance distribution in accordance with the importance scale [76,77]. The intensity of importance is given an odd number from 1 to 9 against its level of importance, where 2, 4, 6 and 8 are intermediate values. 

We assume the matrix values based on experts’ understanding, however, that is uncertain, so, for this reason, we check it for consistent results. If other values are to be assumed, this should follow the same constraints of AHP pairwise comparison matrix and would yield the same results. The steps taken to perform the numerical analysis of the AHP are as follows:

1. Construct the pairwise comparison matrix. The pairwise comparison matrix of attributes is given by:

(11) $$A=\;\left[\begin{array}{ccc} a_{11} & \cdots & a_{1m}\\ \vdots & \ddots & \vdots \\ a_{m1} & \cdots & a_{mm} \end{array}\right]$$

where *a_ij_* (*i,j=1,2,…,m*) represents the importance of the *i-th* index compared to the *j-th* index and *a_ii_=1, a_ji_=1/a_ij_*.

2. Calculate the weight index to check the consistency of the pairwise comparison matrix.

(12) $$W\;=\left[\begin{array}{c} w_{1}\\ \ldots \\ w_{m} \end{array}\right],\;\mathrm{wj}\;=\frac{\sum_{j=1}^{m}(a_{ij}/\sum_{i=1}^{m}a_{ij})}{m}\;$$

3. Calculate the consistency check index as:

(13) $$\mathrm{CI}\;=\;\frac{\lambda_{\max-\;m}}{m-1},\;\lambda_{max}=\frac{1}{m}\sum_{j=1}^{m}\frac{{\left(Aw\right)}_{j}}{w_{j}}$$

where λmax is defined as the maximum eigenvalue. The closer the value of CI to 0, the better the consistency of the pairwise matrix is. Then, to judge on the system consistency, we calculate the revised consistency indicator CR = CI/RI. The values of such indicators are consistent if and only if CR < 0.1. The RI is the average random consistency index and is obtained from a scale that depends on the size of the matrix as shown in Table 1.

According to the pre-described application classes, the matrix is composed of four attributes, which results in a value of RI to be 0.96. 

#### 2.2.4. MADM Algorithms Illustration

With the verities of MADM algorithms, we have shortlisted three potential algorithms to find the most suitable one that achieves the desired goal of our research. 

The main advantages of SAW are its simplicity and low complexity. The aggregation technique in SAW approach makes no difference between cost and benefit kind criteria. Therefore, cost kind criteria ought to be transformed into benefit kind standards at some point of normalization. Thus, a parameter can be outweighed with another parameter, which leads to a rank reversal phenomenon. Formerly, this shape of transformation was often noted as a weakness of the SAW method.

The TOPSIS method is widely used in some models of MADM because this method has several advantages. TOPSIS concept is simple and easy to understand, its computing is efficient and has the ability to measure the relative performance of decision alternatives in simple mathematical form. Its main disadvantages are that it does not do anything about weight stimulating and consistent validation of decisions.

The AHP is the most commonly used solutions to MADM, which have been introduced, in different research areas. AHP is a MADM method, which builds a hierarchy relationship between attributes and alternatives. AHP is using a consistency check to verify the consistency of the pairwise comparison matrix. Using pair comparisons can enable judgments to weight coefficients and easily compare solutions. It is robust and can easily implement and can be adapted in size due to its hierarchical system approach. The main issues among the three algorithms, AHP, TOPSIS, and SAW, are illustrated in Table 2.

The upcoming simulation and analysis section approves the comparison between the three systems; the proposed system model section provides an explanation for our recommendation of using the AHP algorithm to solve the unequal cost load balancing in this paper.

## 3. Proposed System

This paper considers efficient load balance for application data over multiple connections. The solution proposed in this work address are:

- A general solution that is by making a fair distribution of application data over the available multiple connections in accordance with their own preferences.
- Attainment of a load balance solution without altering neither the hardware nor any of TCP/IP protocol suite.

Load-balance techniques can be classified into equal cost load balance (ECL) and unequal cost load balance (UCL). The ECL is based on sending data in an equal distribution form either as per packet or session. The UCL is based on sending data unequally over all available connections.

The load-balance technique uses scheduler modes, either as a round robin or a weighted round robin mode [40]. The round robin sends data as a packet per packet over all available connections in a rotational order. Weighted round robin does nothing different, but its distribution ratio depends on the bandwidth of the connections. The round robin mode has issues related to performance degradation of the TCP applications and multimedia applications. However, the weighted round robin techniques have proven to be relatively better. 

Our proposed model will provide utilization of the available network connections based on the weighted round robin mode. One primary issue with weighted round robin is that it depends on the connection bandwidth. The connection bandwidth is not the seldom attribute used to judge on the connection. One way to overcome this problem is by allowing the proposed model to be dependent on several attributes. The connection utility function describes the mechanism used to distribute the application data as a weighted round robin. 

### 3.1. The Connection Utility Equation

The connection utility equation provides the mathematical representation that is used to utilize the application data as a weighted round robin. In fact, there are several approaches to represent the relation between the application, connections, and device status. An approach is devised in the proposed model from which the connection utility equation is derived.

Assume that the interface utility function is represented in a vector relation between *U* that is the satisfaction level of application *j* (*U_j_*) and *Q* that is the battery consumption for application *j* using interface *i* represented in the vector (*Qj*) [78]. Then, the vector *I_j_* is given by:

(14) $$\left[\begin{array}{c} I_{1j}\\ \ldots \\ I_{nj} \end{array}\right]=\;\alpha_{j}\;\left[\begin{array}{c} U_{1j}\\ \ldots \\ U_{nj} \end{array}\right]+\;\beta_{j}\left[\begin{array}{c} Q_{1j}\\ \ldots \\ Q_{nj} \end{array}\right]$$

Interface utilization may also be achieved by distributing the application data traffic among available connections. The data traffic represented as *W* should be distributed over *m* interfaces for *j* iterations, starting from a single interface until the last *m* interface. This data traffic has unity distribution given by:

(15) $$\sum_{j=1}^{m}w_{j}=\;1$$

Actually, Equation (15) maintains the packet distribution using the equal cost load balance, which achieves the distribution in round robin format. If we assume unequal cost load balance to transmit application data among all connections, the constraint given in (15) would be modified to be:

(16) $$\sum_{j=1}^{m}\alpha_{j}\;*\;w_{j}\leq 1$$

where **α_j_** is the connection utility factor (rate adaptation factor) that is used to adapt the interface bit rate, obtained by a suitable algorithm while calculating the weighting value as a cost. It has two values; either 0 in case of operating at low power transmission state; or a given value in accordance with the cost of the corresponding interface weight *W_j_* being the amount of data transfer per *J* connection which will be sent based on the pre-calculated weight of that connection.

In order to reformulate the constraint condition i.e. sum =1, then we add the term β*W_low_ thus, the connection utility equation is modified to:

(17) $$\beta *\mathrm{Wlow}\;+\sum_{j=1}^{m}\alpha j\;*\;wj=\;1$$

where *β* is the transmission rate factor that is used under low power transmission state (assumed to be <15% of the system power). It has two values; which are either zero, in case of operating in good conditions i.e. system power >15%; or another value that represents the cost of the less power consuming connection under low power transmission state. *W_low_* is the amount of the application data transmission per the less power consuming connection.

In normal cases, i.e. running in a good state, the multiple transmissions are maintained by the aid of the good state connection utility equation as:

(18) $$\sum_{j=1}^{4}\alpha \;*\;wj\;=\;1$$

In case of running at low system power, the transmission is maintained over the less power consuming connection. In this case, the low power state utility in Equation (19) is used to maintain the handover mode, by sending the total traffic over the less power consuming connection.

β*W_low_ = 1 (19)

The overall transmission is then formed from the sum of the transmission in normal power state and the transmission in low power state as represented in Equation (17).

### 3.2. The Proposed System Model

**Figure 2:**
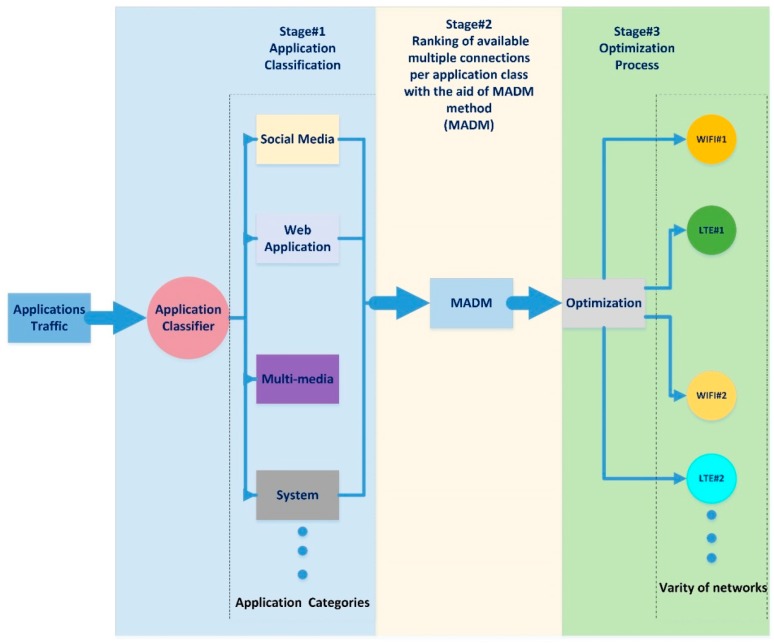
Illustration of the proposed system model.

The proposed system utilizes the application data over available multiple connections with an enhanced weighted round robin technique as a form of unequal cost load balance. The proposed system model consists of three stages, as illustrated in Figure 2.

These proposed model stages in Figure 2 are described in detail in the following subsections. In this proposed model, the application traffic is classified using the classifier module based on defined sets such as social media, web application, multimedia application, system data traffic, etc. [79]. The selected MADM method is used to calculate the priority vector per each classified application class. The optimization process represented in the utility connection equation makes use of the MADM ranking weights. The optimization is done by utilizing the traffic as unequal cost load balance over available connections in an adjustable weighted round robin scheme. We note that the proposed system is characterized by:

- Classifying the application data according to common classes.
- The MADM process, carrying out the ranking process based on the application class.
- Utilizing the available multiple connections with the aid of the utility connection equation as an optimization process by sending the application’s data in an unequal cost load balance scheme.

**Figure 3:**
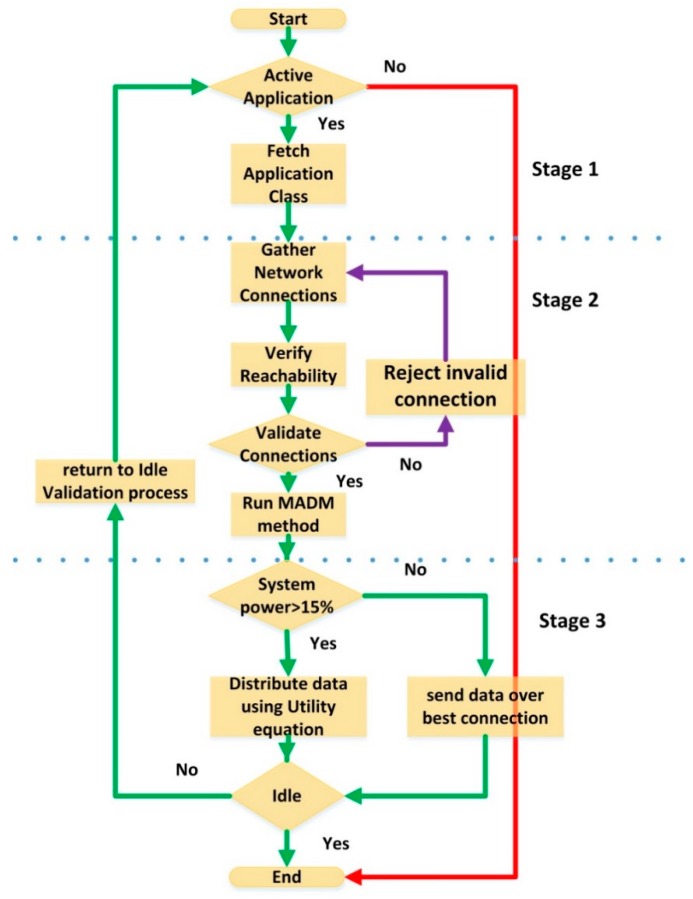
Proposed system flow chart.

For further illustration, the processes that are carried out in the proposed system model can be further explained using the flow chart seen in Figure 3.

The proposed system has two constraints: the system power and the overall path delay. The system power constraint is based on the system running with no less than 15% within its full power state. The constraint is maintained based on a mobile system model, which is beneficial in saving the remaining power state with the most possible application experience. If the system continues with the designed model, then it will lose the remaining power percentage in a short time. For these reasons, we have put the constraint to provide a power saving mode for the device. In case of low system power state, the proposed system will offload the application traffic to the less power consuming connection, i.e. the best ranked interface by the selected MADM method, as defined in Equation (19), until it retains its good system power state for which it will utilize the application data in accordance with Equation (18) [80]. 

The other system constraint is the overall path delay, which is measured by the internet control message protocol (ICMP) probe. The ICMP probe consists of ICMP request and ICMP replay and the probe results represent in the amount of time that the ICMP replay delayed in. The results of the ICMP forms the delay constraint that is used to validate the network connection for not exceeding the 150 ms delay limit [77]. The delay limit was maintained for delay-sensitive applications like multimedia and voice over IP (VOIP). In the process, we relayed on passive probes having less system overhead and providing an estimation for the available multiple connections QoS attributes, which will be stated in simulation results and analysis. 

#### Stage 1: Application Classification

As seen in Figure 3, the application status is defined as either idle or active. In case the application is active, then the process continues, else it will stop. The idle state is checked based on the existence of an open application session. 

Back to the main system model in Figure 2, the distribution of applications data over the available multiple connections in an adaptive weighted round robin scheme based on the application preference requires special treatment. The proposed special treatment is to treat every application in accordance with its preference and needs. In order to do so, it is recommended to provide a division of applications into groups, and each group has an attribute or weight on which the quality of its performance depends. The division is recommended to be performed through a classification stage as intended in this section. The active application session should be classified into one of the following classes with their related favorite attributes [79].

1. Social media: depends mainly on availability which is related to power consumption (PR).
2. Multimedia and gaming: depends mainly on the network delay (D).
3. Web application: depends mainly on speed which is related to throughput (T).
4. System application: this is sensitive to the cost of using the network (C).

Then the process continues to validate the available connections. On the data plan level, the system runs to validate the available network connections based on QoS probing. The network connections are checked against the ICMP echo request and the anticipation of accepted ICMP echo reply value based on the defined delay constraint. 

#### Stage 2: Connection Ranking Using the MADM Method

As seen in Figure 2, a MADM method is required to determine the priority vector as an initial ranking weight of the available multiple connections after which the rule of the utility connection equation is to distribute the application’s data in accordance to the determined weights in unequal cost load balance scheme. The main duty of Stage 2 is ranking the available multiple connections in accordance with the application class and the attributes of the connection.

**Figure 4:**
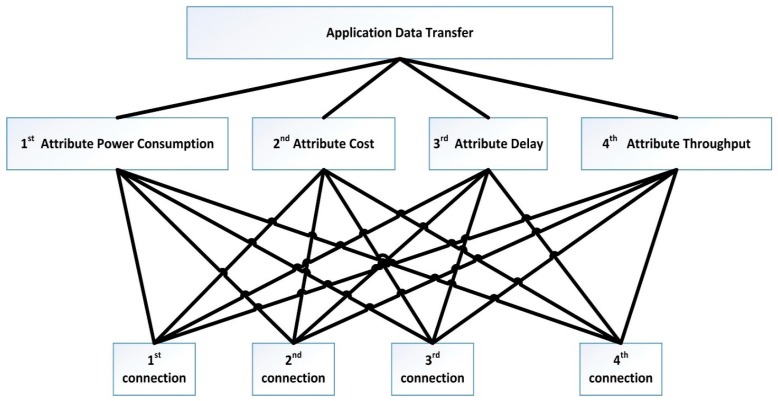
Hierarchy of ranking issue using MADM method.

The hierarchy process is beneficial for the proposed solution. That process conducts a relationship between the applications, the connection attributes influencing the applications, and the available multiple connections which have a different measurement with attributes. As stated in Stage 1, every application has a different dependent attribute for which its performance perform. We use the relationship between the applications, attributes, and available multiple connections to construct the required hierarchy process of MADM method for which it will do the required ranking. As shown in Figure 4 the analysis relationship is illustrated in a hierarchy form. The ranking process yields a weighted metric which will be used by the utility connection equation next to distribute the application data classes over the available multiple connections.

#### Stage 3: Optimization Process

As seen in Figure 2, the optimization process is the last stage of the proposed system model. The application traffic is distributed among available multiple connections by applying overall traffic utilization through the connection utility equation (17). Every application will have a different profile of its own data distribution based on the weighted criteria. The connection utility equation is applying the optimization of the selected MADM ranking process in order to maintain the distribution of the application’s data in accordance with the weighted round robin in adaptive form based on application weighted criteria.

The utility connection equation will use its good state form represented in Equation (18) to distribute the application’s data over available multiple connections, while the system power is in its normal cases. However, it will transit to use its low power state form represented in Equation (19) to offload the application’s data over the less power consuming connection among the available multiple connections, which is determined through Stage 2 of the proposed system. 

### 3.3. Proposed Model Compatibility with Networks Models

**Figure 5:**
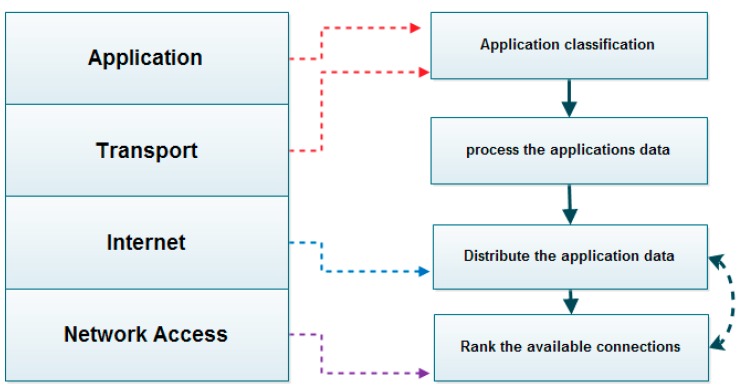
Relationship between the proposed system model and the TCP/IP model.

The proposed model performs unequal cost load balance routing by the distribution of the application traffic among available multiple connections with considering power consumption, application preferences, and QoS parameters. The proposed model is applicable for all network models. However, we have made an illustration of its relationship with the TCP/IP model. The relationship is not dependent on TCP/IP, but it extends to accommodate the IoT model and expands to any model as well. The proposed system model is considered a general solution for the multi-homed devices and terminals, and the TCP/IP relationship was emphasized due to the majority of its implementation. The relationship between the proposed model and the TCP/IP network model is illustrated in Figure 5.

The application and transport layer classify the application’s data into four major classes and assigns their weights. The internet layer provides the unequal cost load balance utilization with the aid of the utility connection Equation (17). The data plane is using a probing based on QoS parameters to confirm the validity of the available network connections according to the TCP/IP network access connection. The outcomes of the proposed model in the form of the distribution of applications data per application class over the available multiple connections are stated next in the model simulation and analysis section.

### 3.4. Distribution of Applications Data Over Available Multiple Connections

The MADM algorithm used to provide the distribution of the application’s data over available multiple connections described by the pseudo-code represented in Algorithm 1. In the algorithm, the *k* network connections are evaluated and verified, we determine the application data that are distributed over the *k* connections with the aid of the utility connection equation. The application traffic is classified and analyzed according to the MADM process. 

**Algorithm 1:** Application based MADM Process.
(1)InitializationA: given k interfaces Ik, 1 < k < KB: given file size N bytesC: given A is the comparison matrix of size n*nD: w is the eigenvector of size n*1E: λmax is the eigenvalue, λmax ϵ R > n.(2)Root of sum-product RSP $=(\overset{n}{\underset{k=0}{\sum }}\overset{n}{\underset{j=1}{\prod }}A\;$)^1/n(3)Weight vector = [ $\overset{n}{\underset{j=1}{\prod }}A$ )^ (1/n) / RSP(4)λmax = ∑(( $\overset{n}{\underset{j=1}{\sum }}A$ ) * Weight Vector)(5)CI = (λmax − n )/(n-1)(6)If CR > 0.1, then(7)Go to (2)(8)Else(9)w = weight vector (10)$\overset{4}{\underset{j=1}{\sum }}\alpha \;*\;wj$ = 1


The numerical calculations of the selected MADM method are explained in detail within the model simulation and analysis section along with a verification of the proposed system for sending applications data per available multiple connections.

## 4. Model Simulation and Analysis

**Figure 6:**
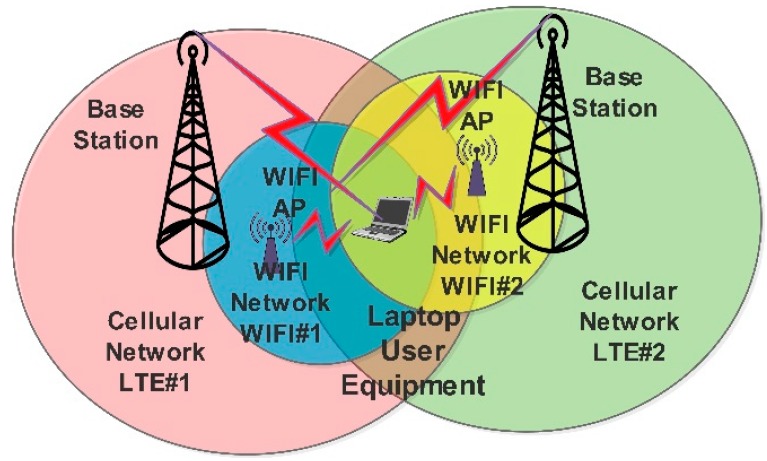
MH in a HetNet scenario.

In order to evaluate the performance of the proposed model, we consider a heterogeneous wireless network that consists of two LTE base stations, two WLAN access points (AP1, AP2), and a user laptop device. Considering the case of WLAN and LTE coexistence that has been studied by a large number of studies in the border literature. We assume that the user device has multi-RAT connections, i.e. heterogeneous network connections, and the networks labeled as networks 1 and 2 correspond to WiFi#1 and LTE#1, respectively, and networks 3 and 4 correspond to networks WiFi#2 and LTE#2, respectively. The device is seeking robust internet connectivity using four simultaneous connections as illustrated in Figure 6. The location of the laptop device that performs the measurements is close to WiFi#1, which has few subscriber devices, and exists near the optimum coverage region of the LTE#1 network, LTE#2 is far from the right-hand side, and WiFi#2 is on the top right direction as referred to in Figure 6 with more subscriber devices.

### 4.1. Simulation Parameters

The connection characteristics/parameters per each available connection are illustrated in Table 3 and have been collected based on the network model that is illustrated in Figure 6 using a specialized site survey software. The connections characteristics were measured based on the related application preference to evaluate the simulation results according to real measurements. The parameters used in the simulation are the cost per connection (C), power consumption per connection (Pr), throughput per connection (T), packet delay per connection (D). In the simulation process, a computer is used with Intel Core i5 quad-core CPU 2450M @ 2.5 GHz, 8 GB RAM.

### 4.2. Simulation Results and Analysis

In this section, we analyze the data collected from the simulation. The simulation is based on MATLAB, which runs the proposed model through an implemented MATLAB M-file code. The selection of MATLAB is based on the objective of illustrating the performance of the proposed system in numerical calculations. The proposed system considers a general situation of using application classes, selected MADM ranking process and utility connection equation, which depends on numerical calculations and MATLAB is fine in that. The simulation runs for four traffic classes namely: social media, multimedia and gaming, web, and system.

#### 4.2.1. Multiple Attribute Decision Making

**Figure 7:**
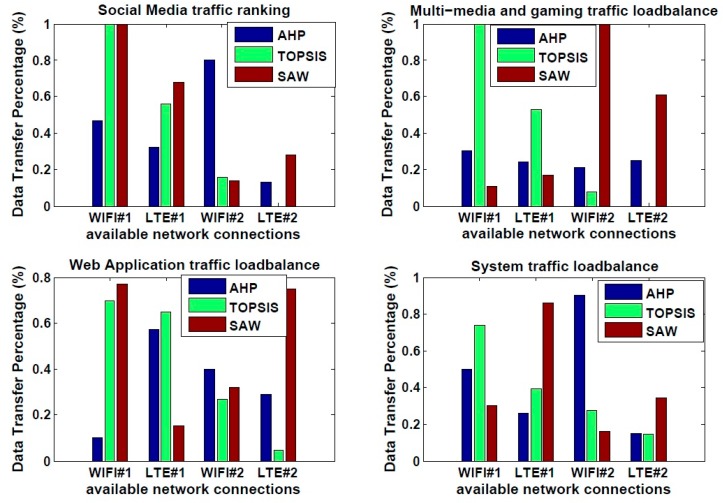
Performance analysis of three MADM methods.

A large number of alternative approaches based on multiple attributes have been devised in the literature to choose from multiple connections. Those approaches are using MADM methods. The AHP algorithm is compared to other algorithms namely, TOPSIS, and SAW to investigate which of them provide a suitable ranking for the proposed system model. To examine the impact of multiple connections ranking, we tested each algorithm against every application class as illustrated in Figure 7.

After evaluating the performance of the three-load balance MADM algorithms based upon different application class, i.e. social media, multimedia-and-gaming, web, and system applications, we can summarize the performance of three algorithms based on Figure 7 as follows:

(a) In case of social media, it is found that is the best algorithm for the load balance solution is the AHP as it provides satisfactory performance over multiple connections, which is beneficial for data distribution, while SAW and TOPSIS are more applicable in traffic handoff or handover solution.

(b) In case of multimedia and gaming, it is found that is the best algorithm for the load balance solution is the AHP as it provides satisfactory performance over multiple connections, which is beneficial for data distribution, while SAW and TOPSIS are more applicable in traffic handoff or handover solution.

(c) In case of the web application, it is observed that is the best algorithms for the load balance solution are the AHP and TOPSIS as both provide satisfactory performance over multiple connections, which is beneficial for data distribution, while SAW is more applicable in traffic handoff or handover solution.

(d) In case of system application, it is deduced that is the best algorithms for the load balance solution are TOPSIS and AHP as they provide satisfactory performance over multiple connections, which is beneficial for data distribution, while SAW is more applicable in traffic handoff or handover solution.

Based on the evaluation of the three MADM methods and the comparison result carried out in the related work section, we may conclude that that AHP is the best decision method for load balance solution over multiple connections. The AHP also has some advantages such as being a hierarchy in process, and the consistency check of its numerical calculations, which make from it relevant to be selected. The evaluation illustrated that the AHP is suitable for load balance solution for its satisfactory ranking distribution. However, TOPSIS and SAW are efficient in handover solution for their ranking based on building close results in the ranking process and build their interest to highlight the best which makes from it more suitable for handover solutions. 

As the AHP is found to be the most suitable MADM algorithm for achieving unequal load balance distribution, it will next be embedded in the proposed system model as the selected MADM method to further test its performance on the attributes selected for various application classes.

#### 4.2.2. Numerical Assumptions of AHP

Before we provide an illustration of the proposed system simulation output we have to explain the numerical assumptions of the AHP as the selected MADM method for the proposed system. The AHP numerical process starts by constructing the pairwise comparison matrix per each application class and the distribution is made according to the relative importance scale and relations discussed earlier in the proposed system section. The numerical distribution of the application classes’ pairwise comparison matrix is assumed as follows.

##### Social Media Application

Using with the power consumption (PR) as an attribute to normalize the distribution matrix. PR is self relatively equally important, PR to C is relatively important, PR to D is relatively very strong important and PR to T is relatively in a moderate importance relationship. Also, C to D has relatively strong importance and C to T is relatively moderate important. D is relatively important with respect to T. The pairwise of the social media application class is illustrated in Table 4.

##### Multimedia and Gaming

The usage of the delay (D) as an attribute to normalize the distribution matrix. D is relatively moderate, very important with respect to PR, very important with respect to C, self-equal importantly, and relatively more important than *T*. C is relatively important than the PR. T is relatively moderate very important to D and is relatively important to C. The pairwise of the multimedia and gaming application class is illustrated in Table 5.

##### Web Application

The throughput (T) is used as an attribute to normalize with it. T is the relatively more important than D, T is relatively moderate, very important with respect to C, T is relatively moderate important in respect with PR, T is self relatively equal important. PR is relatively important with respect to C. PR is relatively moderate very importantly in respect with D. D is relatively moderate very importantly in respect with C. The pairwise of the web application class is illustrated in Table 6.

##### System Application

The cost (C) is used as an attribute to normalize the distribution matrix. Cost is the most important attribute. C is self relatively equal important. C is relatively important with respect to PR. C is relatively moderate very important with respect to D. C is relatively very important with respect to T. D is relatively moderate important with respect to T. T is relatively moderate very important with respect to PR. The pairwise of the system application class is illustrated in Table 7.

The numerical calculations of the AHP for the construction of the pairwise comparison matrix are performed by maintaining the importance of degrees based on the application QoS. The numerical calculations as well are validated with the AHP consistency check. Then, the AHP ranking weights will be used as a cost metric to be utilized using the utility connection equation. 

#### 4.2.3. Utilization of Multiple Connections Using Utility Connection Equation and AHP

**Figure 8:**
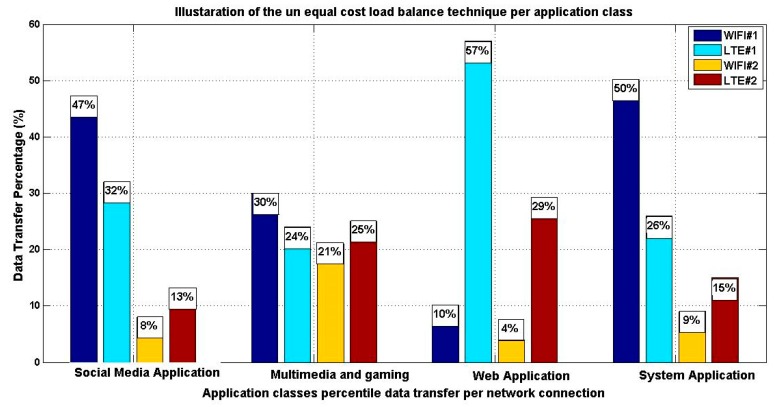
Illustration of the unequal cost load balance per application class using AHP.

The Algorithm 1 Matlab M-file is used to analyze the performance of the proposed solution by providing unequal distribution of the application data using both the AHP as the selected MADM method and the proposed utility connection equation. As seen in Figure 8, simulation results reveal that the proposed model achieves the required objective, which is transmitting the data traffic per each application preference over the available multiple connections as an unequal cost load balance scheme instead of offloading application data based on the ranking of connections. As seen in Figure 8, the results of the proposed model processed by Algorithm 1 show a variation in application data transfer as a reflection of their requirements. Moreover, and with reference to the measured data represented in Table 6 and the relevant application class results, we deduce that they are compatible and proved the efficiency of the proposed optimization process.

**Figure 9:**
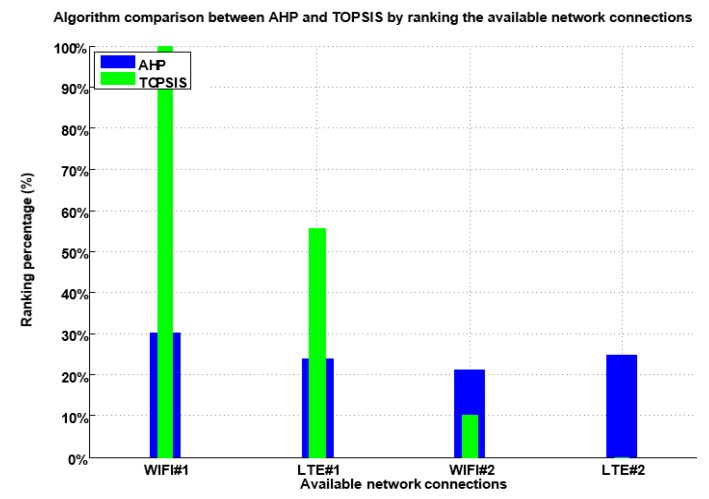
Comparison of network connections utilization based on the proposed model versus the TOPSIS algorithm.

The varieties of the applications data distribution were significantly better in term of the application preference and which is based on their relative connection attribute and needs. Figure 9 illustrates the comparison between the proposed model and utilizing the TOPSIS algorithm as an application based handover solution. The comparison is based on the multi-media application class. As shown from this figure, the improvement is represented in better utilization of the application data traffic over the available network connections which is translated into better utilization of the total throughput and less usage of the interface buffer. 

It is observed that WiFiI#1 is the best network in both algorithms. However, there is a significant difference in ranking other networks. AHP ranks the available network connections in an integral form, which aids in distributing the application data over multiple networks in unequal cost load balance scheme. The proposed model addresses the usage of all available connections without any exclusion. As a comparison of handover solution, we can see that load balance provides better transmission in terms of fair distribution of data over multiple connections and which aims at boosting the overall performance. Moreover, in case of low system power, the proposed model transforms into a handover solution and transmits over the less power consumption connection (WiFi#1) like TOPSIS. The proposed system model checks the end device system power against the low power constraint, which is 15% of the total system power. The proposed system is considering the mobile energy system model for power criteria and estimation. At low system power state, the system model stops the load balance technique and perform handover. The AHP module is still in effect as it is used to rank the available connections and the proposed system use the highest ranked connection to perform handover through it. The applications are processed in general mode, based on social media application class processing for which the dependency is based on power consumption. So in case of low power, the proposed system will transmit all applications data over the less power consuming connection. Based on our calculations, the proposed system will offload all applications data over WIFI#1 as it is the less power consuming interface. The system will retain its normal unequal cost load balance based on application class as the system power is above the low power state.

The proposed system assumes classification of data, which requires a classifier module and the proximity of this module may be in a miss due to heavy application load. The AHP numerical distribution is static and designed based on relative importance degree of the attributes, and changing those values may yield a different result. The mitigation for that already exists with the aid of the utility connection equation which settles the ranking priorities into an adequate ratio used as a distribution ratio of the application data. Despite the separate controller that is used to control and manage the end terminal traffic, it has some concerns. These are represented in being a single point of failure and a bottleneck for the network traffic processing, the extra propagation, and processing delay that exist between controlled MH and its controller.

## 5. Future Work

Future studies could investigate the integration between the proposed model and SDN through a real scenario experiment. The SDN will aid in providing a dynamic classification of applications and dynamic propping of network connections. Also, the SDN will aid in constructing the pairwise comparison matrix and AHP computation with the multi-homed host. Besides, the merits of the SDN controller, which is used to sync with the data plane module. The future model is considered a hybrid model that is made use of the user-centric and the network-centric merits.

## 6. Conclusions 

The proposed model provided a novel user-centric scheme for multihoming in heterogeneous wireless networks as unequal cost load-balance. The relationship between multihoming and TCP/IP model was explained and noted that it could be extended to accommodate the IoT model, adding that it could expand to any model as well. This paper examined some of the most notable algorithms for multiple attributes decision making MADM used in alternative decision making for providing a suitable solution for an unequal cost load-balance with multiple criteria, such as SAW, TOPSIS, and AHP. Comparing MADM algorithms, it was found that in case of applications types such as social media and multimedia and gaming, AHP was the most suitable MADM algorithm for achieving unequal load balance distribution, while SAW and TOPSIS were more applicable in traffic handoff or handover solution. In the case of the web and system application, both AHP and TOPSIS provided satisfactory performance over multiple connections, which was beneficial for data distribution, while SAW was more applicable in traffic handoff or handover solution. Thus, it was recommended that AHP was the best decision method for load balance solution over multiple connections. AHP performance along with the connection utility equation was examined on attributes selected for various application classes and simulation results revealed that the proposed model achieved the required objective instead of offloading application data based on the ranking of connections. As a comparison of handover solution, it was concluded that load balance enabled a better transmission in terms of fair distribution of data over multiple connections. The proposed system considered the mobile energy system model for power criteria and estimation. Moreover, it provided an efficient energy consumption for saving system power in critical power status. In the case of low system power, the proposed model transformed into a handover solution and transmitted over the less power consumption connection.

## Figures and Tables

**Table 1:** Values of RI based on matrix dimension.

**Dimension**	1	2	3	4	5	6	7
**RI**	0	0	0.58	0.96	1.12	1.24	1.32

**Table 2:** AHP, TOPSIS, and SAW algorithms illustration.

	AHP	TOPSIS	SAW
Advantages	Can include qualitative criteria	Simple to employ	Simple to employ
Disadvantages	With subjectivity	Ranking abnormality, optimal the solution depends on the vector Position of alternatives	Issues with weights, ranking identification
Consistency	Yes	No	No
Core Process	Hierarchy principle	Distance principle	Weighted average principle

**Table 3:** Characteristics of alternative connections.

Connection	Cell Radius (meter)	Power Consumption (Mille Watt)	Cost ($)	Delay (Mille sec.)	Max Capability (Kbps)	Throughput (Kbps)
WIFI#1	65	1.8	7	50	6000	350
LTE#1	415	2.3	10	85	10000	6800
WIFI#2	45	3	15	90	8000	210
LTE#2	340	2.5	11.5	70	15000	3680

**Table 4:** The pairwise comparison matrix for the network attributes for social media applications.

Social Media	PR	C	D	T
**PR**	1	2	7	3
**C**	1/2	1	5	3
**D**	1/7	1/5	1	1/2
**T**	1/3	1/3	2	1

**Table 5:** The pairwise comparison matrix for the network attributes for multimedia and gaming applications.

Multimedia and Gaming	PR	C	D	T
**PR**	1	1/2	1/7	1/6
**C**	2	1	1/7	1/4
**D**	7	7	1	2
**T**	6	4	1/2	1

**Table 6:** The pairwise comparison matrix for the network attributes for web applications.

Web Application	PR	C	D	T
PR	1	3	4	2
C	1/3	1	1/5	1/6
D	1/4	5	1	1/7
T	1/2	6	7	1

**Table 7:** The pairwise comparison matrix for the network attributes for system application.

System Application	PR	C	D	T
PR	1	1/3	4	1/5
C	3	1	6	7
D	1/4	1/6	1	2
T	7	1/7	1/2	1
